# Supplementary material for: Immediate or Delayed Transplantation of a Vein Conduit Filled with Nasal Olfactory Stem Cells Improves Locomotion and Axogenesis in Rats after a Peroneal Nerve Loss of Substance
Source: Int J Mol Sci. 2020 Apr 11;21(8):2670. doi: 10.3390/ijms21082670 (PMC7215801; doi:10.3390/ijms21082670)
Supplement: Supplementary file 1 [file ijms-21-02670-s001.pdf]

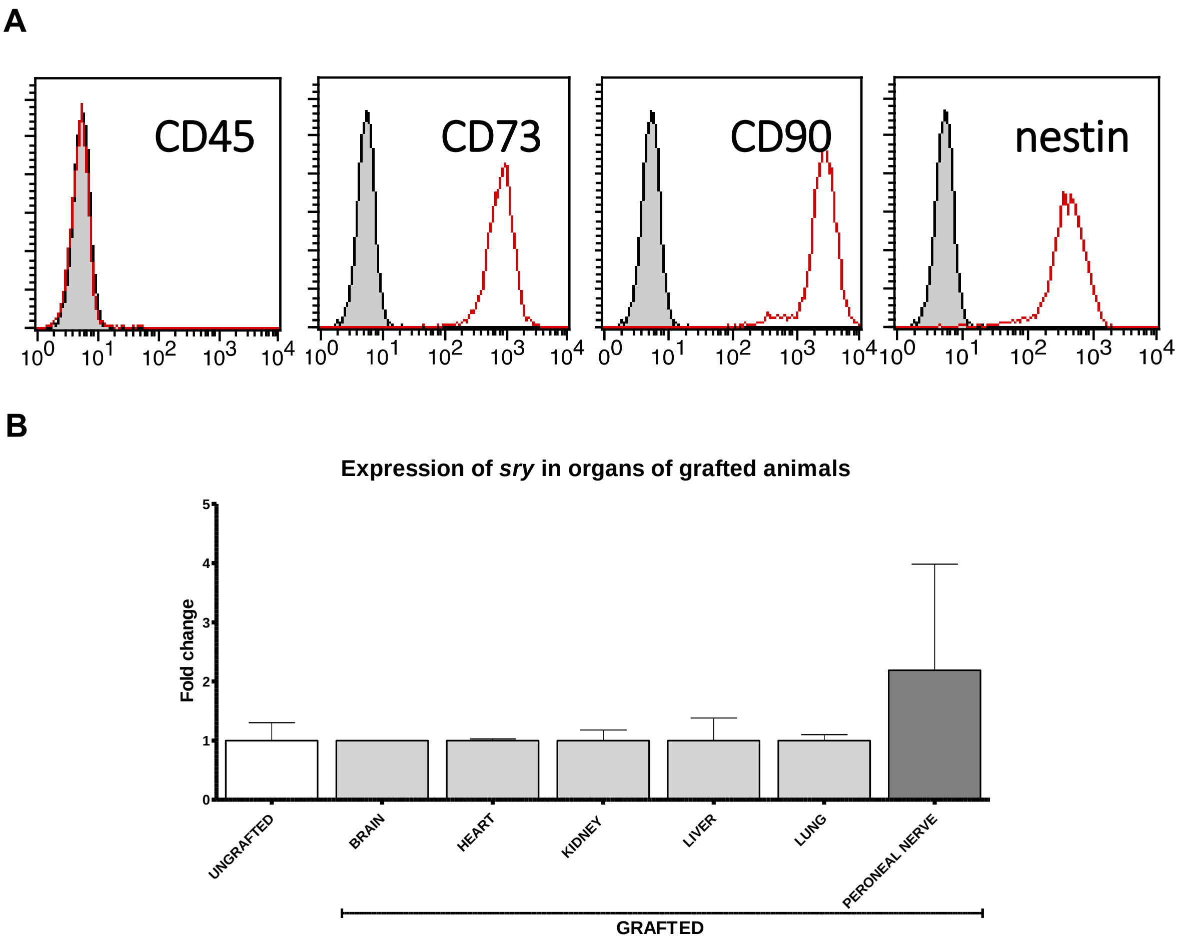

Supplementary figure 1. *Cell characterization before grafting and stem cell distribution, 3 months post-surgery*. **A.** Most of the cultivated cells are positive for CD73, CD90, nestin and negative for CD45, indicating that they express the recognized olfactory stem cell markers. **B.** Male purified stem cells were grafted into female hosts. The presence of male cells into the peroneal nerve, brain, heart, kidney, liver and lung was assessed using the quantitative PCR. The expression of the gene *sry* (sex determining region of Y chromosome) was solely found in the grafted nerve.
